# Epidemiology of intestinal parasitic infections in preschool and school-aged Ethiopian children: a systematic review and meta-analysis

**DOI:** 10.1186/s12889-020-8222-y

**Published:** 2020-01-28

**Authors:** Legese Chelkeba, Zeleke Mekonnen, Yonas Alemu, Daniel Emana

**Affiliations:** 1 Department of Clinical Pharmacy, School of Pharmacy, College Health Sciences, Jimma University, Jimma, Ethiopia; 2 Jimma University Medical center (JUMC), Jimma, Ethiopia; 3 Department of Parasitology, School of Medical Laboratory Sciences, College Health Sciences, Jimma University, Jimma, Ethiopia

**Keywords:** Preschool-age children, School-age children, Intestinal parasites, Ethiopia, Meta-analysis

## Abstract

**Background:**

Numerous studies have been carried out on assessing the prevalence of intestinal parasites infections (IPIs) amongpreschool and school-age children in Ethiopia, but there is lack of study systematically gathered and analyzedinformation for policymakers. Therefore, the aim of this systematic review and meta-analysis was to provide a summary on prevalence, geographical distribution and trends of IPIs among preschool and school-age childrenin Ethiopia.

**Methods:**

The search were carried out in Medline via PubMed, Scopus, Science Direct, Web of Science, and Google Scholar from 1996to July2019 for studies describing prevalence of IPIs among preschooland school-age children. We conducted meta-regression to understand the trends and the source of heterogeneity and pooled the prevalence using ‘metaprop’ command using STATA software version 14.

**Results:**

Eighty-three(83) studies examining 56,786 fecal specimens were included. The prevalence of IPIs was 48%(95%CI: 42 to 53%) and showedsignificantly decreasing trends 17% (95% CI: 2.5 to 32%) for each consecutive 6 years) and was similar in males and females. The pooled prevalence in years 1997–2002, 2003–2008, 2009–2014 and > 2014 was 71% (95% CI: 57 to 86%), 42% (95% CI: 27 to 56%), 48% (95% CI: 40 to 56%) and 42% (95% CI: 34 to 49%), respectively. Poly-parasitism was observed in 16% (95% CI: 13 to 19%,) of the cases.

**Conclusion:**

Intestinal parasite infections are highly prevalent among preschool and school-age children and well distributed across the regional states of Ethiopia. Southern and Amhara regional states carry the highest burden. We observed significant decreasing trends in prevalence of IPIs among preschool and school-ageEthiopian children over the last two decades. Therefore, this study is important to locate the geographical distribution and identified high risk areas that should be prioritized further interventions, which complement global efforts towards elimination of IPIs infections by 2020.

## Background

Parasitic infections caused by intestinal helminths and protozoan are among the most prevalent infections in developing countries carrying high burden of morbidity and mortality in these areas [1]. Specifically, economically disadvantaged children living in tropical and sub-tropical regionswith a limited or no access to safe drinking water, inadequate sanitation, and substandard housing are the most affected ones [2]. Epidemiological evidence suggests that an estimated over one billion people in the world, majorly children were infected with intestinal parasites caused by helminths and protozoa [3]. Majority of the infections were due to Ascaris lumbricoides, hookworm, and Trichuris trichiura [4, 5]. More than 267 million preschool-age children and 568 million school-age children live in areas where these parasites are intensively transmitted [6]. *Cryptosporidium* species, *Entamoeba histolytica* and *Giardia duodenalis* were the most common protozoan infections in children under 5 years in sub-Saharan Africa [7].

The regional distribution and prevalence differences of IPIs among children are mainly due to differences in degree of fecal contamination of water and food, climatic, environmental and socio-culture [8–10]. The prevalence among under-five, preschool and school children were reported as 17.7% in Riyadh, Saudi Arabia [11], 52.8% in an urban slum of Karachi, Pakistan [12], 19.6% in Zambia [13] and 30% in Khartoum, Sudan [13]. In Ethiopia, prevalence varies across the regions in the country. For instance, the prevalence was 85.1% in Wondo Genet (Southern region) [14], 48.1% in Aynalem village (Tigray region) [15], 17.4% in Debre Birhan (Amhara region) [16], 26.6% in Hawassa (Southern region) [17], 24.3% in Wonji Shoa Sugar Estate (Oromia region) [18], 18.7% in Woreta (Amhara region) [19], 25.6% in Dembiya (Amhara region) [20] and 41.1% in Jimma town (Oromia region) [21].

School-agechildren are the most affected ones due to their habits of playing or handling of infested soils, eating with soiled hands, unhygienic toilet practices, drinking and eating of contaminated water and food [22]. Intestinal parasite infections lead to malnutrition, mal-absorption, anemia, intestinal obstruction, mental and physical growth retardation, diarrhea, impaired work capacity, and reduced growth rate constituting important health and social problems [10, 18, 23, 24].

Numerous epidemiological studies have been performed on assessing the prevalence of IPIs among children in Ethiopia, but there is lack of systematically gathered and analyzed information for policymakers. Therefore, the aim of this systematic and meta-analysis was to provide a summary on prevalence, geographical distribution and trends of IPIs among preschool and school-age children in Ethiopia.

## Methods

### Search strategy and data extraction

The search were carried out in Medline via PubMed, Scopus, Science Direct, Web of Science and Google Scholar using searching terms intestinal parasite infection” OR “helminths” OR “protozoa” AND “Ethiopia”. Searching was carried out on articles published from 1997 to March,2019 and limited human studieswith language restriction to English. A manual search for additional relevant studies using references from retrieved articles and related systematic reviews was also performed to identify original articles we might have missed. Conference abstracts and unpublished studies were excluded. We did our analyses according to the Preferred Reporting Items for Systematic Reviews and Meta-Analyses (PRISMA) statement [25].

### Participants, inclusion and exclusion criteria

Two authors independently (LCH&DE) assessed the inclusion criteria and disagreement was solved by discussion with the third author (ZM). We included the studies if they met the following criteria: the study design was an observational study (prospective cohort, case-control, retrospective cohort, or cross-sectional) or controlled clinical trial which documented the baseline prevalence or incidence of IPIs. We included all studies reported the rateor proportion of IPIs in preschool and/or school-age children or both. We excluded studies reporting case reports, case series, studies that compared the sensitivity and specificity of different methods used for diagnosis of intestinal parasites and studies not reported either prevalence or incidence of IPIs as outcome of interest. The terms preschool and school-age children were defined according to the original studies. Accordingly, preschool-age children were defined aschildren of age below 5 years while,school-agechildren were children of age 5 and above. Poly-parasitism was defined as concurrent infection with different species of intestinal parasites either helminths or protozoa.

### Data extraction and quality assessment

The two authors (LCH and DE) defined protocol for data extraction and assessed them independently for eligibility and disagreements were resolved by discussion with the third author (ZM). We extracted information on name of the first author and year of publication, study design, population studied (preschool age children, school age children or both), gender, region & sites of study, Method (s) for identification of the parasites, total sample size and the number of the positives (percentage). The Grading of Recommendation Assessment, development and Evaluation (GRADE) approach was used to assess the overall quality of evidence [26]. Accordingly, studies were given one point each if theyhad probability sampling, larger sample sizes of more than 200, and repeated detection and up to four points could be assignedto each study. Publications with a total score of 3–4 points were considered as high quality, whereas 2 points represented moderate quality and scores of 0–1 represented low quality.

### Statistical analysis

We used forest plots to estimate pooled effect size and effect of each study with their confidence interval (CI) to provide a visual summary of the data. A random-effects model was used in this meta-analysis because of anticipated heterogeneity. All reported *P* values were 2-sided and were statistical significant if *P* < 0.05. Statistical heterogeneity among studies was expressed as the *P* value (Cochran’s Q statistic), where a *P* < 0.05 and I^2^ ≤ of 25–50% were considered as low heterogeneity and I^2^ > 50% indicated substantial heterogeneity. We also used Begg’s Funnel plot and Egger’s regression test for evaluating the possibility of publication bias. A potential source of heterogeneity was investigated by subgroup andmeta-regression analysis. The factors included were geographical regions and cities of Ethiopia, age of children (preschool vs. school-age children), and years of publication (1997–2002, 2003–2008, 2009–2014 and > 2014). We conducted meta-analysis using ‘metaprop’ commandusing STATA software, version 14, STATA Corp,College Station, TX.

## Results

### Literature searches and selection

**Fig. 1:**
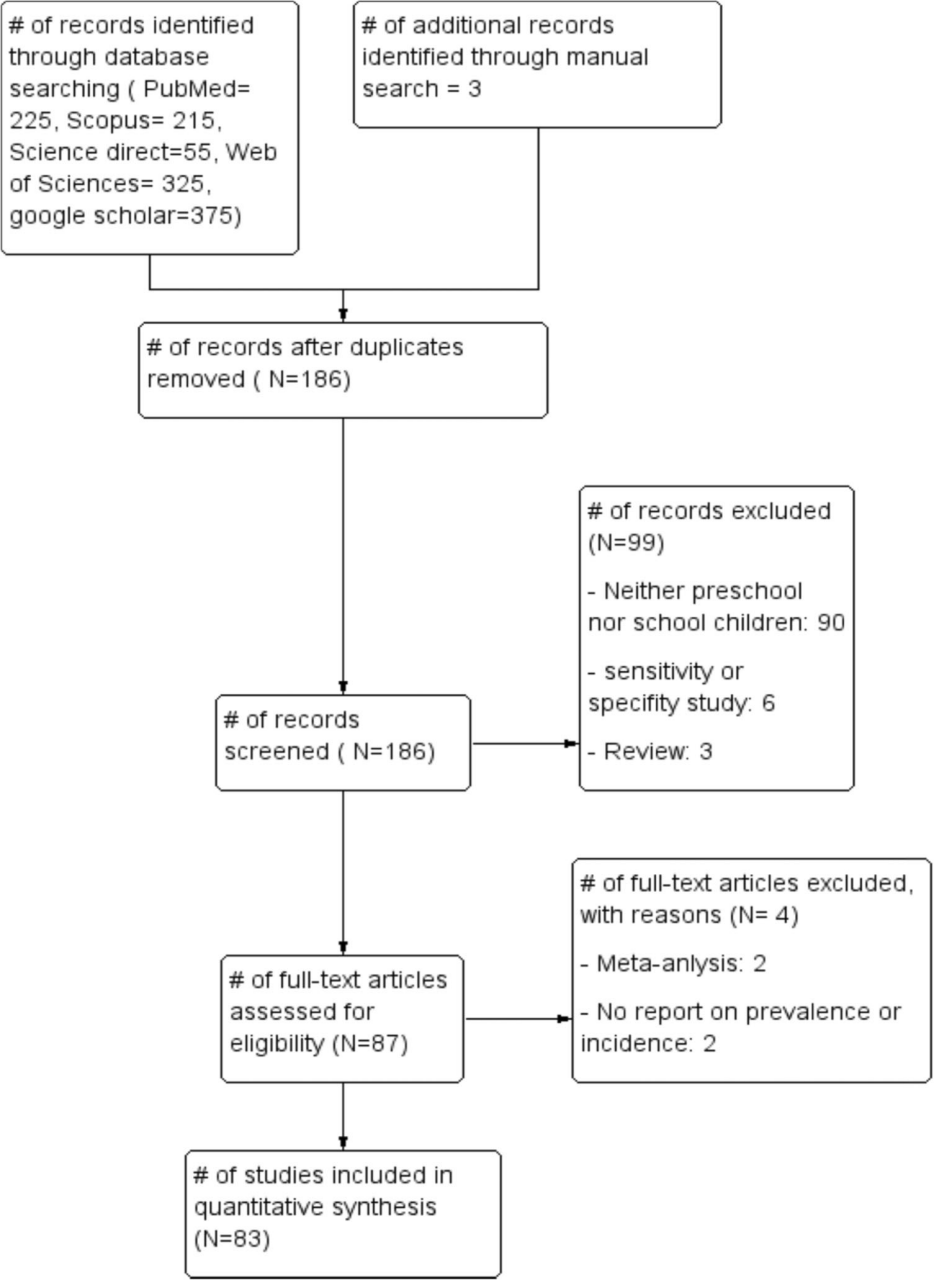
Flow diagram showing the selection process

We identified systematically 1198 publications, of which 83 were eligible for inclusion in the final analyses. The details of our search strategy were depicted in Fig. 1. Our initial search of electronic databases such as Medline via PubMed, Scopus, Science direct, Web of Sciences and Google scholar yielded 1195 articles and 3 articles manually from which 186 records remained after removing 1012 duplications. Up on screening the articles, 99 articles were further excluded; 90 were irrelevant because they were not specifically about preschool or school-age children, 6 studies were about sensitivity and specify of diagnosis of IPIs, 3 articles were review articles. Up on further access to the full texts of 87 articles, 4 were excluded for the following reasons; 2 were meta-analyses and 2 articles lacked outcome of interest. Finally, 83 published between 1997 and 2019 fulfilling the inclusion criteria were included in the analyses.

The sample size of the included studies ranged from 100 [27] to 15,455 [28] with a total number of 56,786 participants [14, 16, 17, 21, 24, 27–103]. Most of the studies were reported from Amhara regional 33(40%) followed by Oromia region 21(25%). The rests were reported from South region 18(22%), Tigray region 9(11%), Benishangul-Gumuz region 1(1%) and Addis Ababa city 1(1%). With regard to the study design, majority of the studies were cross sectional in design (79 studies), 2 were controlled clinical trials, 2 were prospective follow up cohort studies and 1 was case-control. Sixty six studies were about IPIs in school-age children, 13 were in preschool-age children (under-five) and 4 were studies involved both preschool and school-age children. According to our quality assessment criteria, 34 publications were of high quality with a score of 3, 12 had a score of 2 indicating moderate quality and the remaining 37 were of low quality with a score of zero or one [Table 1]. Prevalence estimate and heterogeneity analysis.

**Table 1:** Characteristics of the 83 eligible studies of intestinal parasite infections in Ethiopia

Author	Study design	Population	Male	Female	Study site (s)Region	Methods	No. sample	No. positive (%)	Quality assessment
Degarege 2013 [40]	Cross-sectional	School children	187	216	Tikur Wuha, Gojam,Amhara region	Kato-Katz	403	235 (58.3%)	2
Abdi 2017 [75]	cross-sectional	School children	207	201	Zegie Peninsula, Gojam, Amhara region	Formalin-ether	408	282 (69.1%)	3
Abera 2014 [76]	Cross-sectional	School children	193	192	Bahir Dar, Amhara region	Formal-ether	385	170 (44.2%)	3
Wegayehu 2013 [51]	Cross-sectional	School children	191	193	GirarJarso and Dera, North Shewa Zone, Oromia Region	Direct and formalin- ether and modified Ziehl-Neelsen	384	81(21.1%)	2
Amare 2013 [35]	Cross-sectional	School children	218	187	Gondar town, Amhara region	Direct, formal-ether and Kato-Katz	405	92(22.7%)	3
Gelaw 2013 [45]	cross-sectional	School children	170	134	University of Gondar Community School, Amhara region	Direct and formol-ether	304	104 (34.2%)	3
Abossie 2014 [78]	Cross-sectional	School children	191	209	GamoGofa Zone, South region	Direct and formol-ether	400	324(81.0%)	3
Mathewos 2014 [57]	Cross-sectional	School children	139	122	Gorgora and Chuahit towns, Gondar, Amhara region	Direct, formol-ether and modified Ziehl-Neelsen	261	174 (66.7%)	2
Gizaw 2018 [20]	Cross-sectional	Preschool children	106	119	Dembiya, Gondar Zone, Amhara region	Kato-Katz	225	58(25.8%)	3
Wegayehuet 2016 [77]	Cross-sectional	Both	154	132	Holetta, Sendafa and Chancho, Oromia region	PCR	312	48(16.8%)	2
Yimam 2016 [74]	Cross-sectional	School children	187	216	Tikur Wuha Elementary School, Amhara region	Formol-ether and Kato-Katz	403	235(58.3%)	3
Hailegebriel 2017 [79]	Cross-sectional	School children	177	182	Dona Berber, Bahir Dar, Amhara region	Formol-ether	359	235 (65.5%)	3
Alemu 2018 [80]	Cross-sectional	School children	196	195	ArbaminchZuria, South region	Formol-ether	391	182(46.5%)	2
Alemu 2019 [82]	Cross-sectional	School children	180	171	Birbir town GamoGofa, South region	Direct and formol-ether	351	95 (27.1%)	3
Mekonnen 2019 [19]	Cross-sectional	Preschool children	152	158	Woreta health center, Gondar, Amhara region	Direct and Kato-Katz	310	58 (18.70%)	3
Jejaw 2015 [41]	Cross-sectional	School children	228	232	Mizan-Aman town Bench Maji, South region	Direct and formol-ether and Kato-Katz	460	353 (76.7%)	3
Alemu 2016 [81]	Cross- sectional	Preschool children	183	218	Dembiya District, Gondar, Amhara region	Kato-Katz	401	141 (35.2%)	3
Alemayehu 2017 [58]	Cross-sectional	School children	287	216	Wolaita Zone, South region	Kato-Katz and formalin-ether	503	363(72.2%)	3
Gashaw 2015 [61]	Cross-sectional	School children	255	295	Maksegnit and Enfranz Towns, Gondar, Amhara region	Kato-Katz	550	365(66.4%)	3
Bajiro 2016 [83]	Cross-sectional	School children	238	262	Jimma town, Oromia region	Kato-Katz	500	120(24%)	3
Amor 2016 [84]	Cross-sectional	School children	225	171	Rural area of Bahir Dar, Amhara region	Formol ether	396	327(82.6%)	3
Nute 2018 [28]	Cross-sectional	School children	418	8037	Ten zones of the Amhara region	Formol ether	15,455	5626(36.4%)	3
Zemene 2018 [16]	Cross-sectional	Preschool children	118	118	DebreBirhan hospital, North Shewa, Amhara region	Direct and the formol-ether	247	43 (17.4%	1
Gebretsadik 2018 [85]	Cross sectional	Preschool children	133	99	Dessie referral Hospital, Amhara region	Direct, formol-ether and modifiedZiehl-Neelsen	232	36 (15.5%)	1
Mulatu 2015 [17]	Cross-sectional	Preschool children	81	77	Adare Hospital and Millennium Health Centre, Hawassa, South region	Direct, formol-ether and modifiedZiehl-Neelsen	158	42 (26.6%)	3
Bekana 2019 [86]	Cross-sectional	School children	172	145	Guma and YachiYisa in Gomma, Jimma, Oromia region	Kato-Katz and formol-ether -	317	224 (70.4%)	3
Diro 2015 [87]	prospective cohort	Both	85	37	University of Gondar and Kahsay Abera Humera hospitals, Amhara region	Direct, formol-ether and Kato-Katz	122	58(47.5%)	1
Birhanu 2018 [88]	cross sectional	School children	194	228	Pawe Town, Benishangul-Gumuz Region	Direct	422	130 (30.8%)	1
Fentie 2013 [32]	Cross-sectional	School children	361	159	Lake Tana Basin, Amhara region	Kato–Katz and formol-ether	520	371(71.3%)	3
Aiemjoy 2017 [66]	Cross-sectional	Preschool children	NA	NA	GonchaSisoEnese, Gojam, Amhara region	Formol-ether	212	138 (65.1%)	2
Desalegn 2014 [31]	Cross-sectional	School children	271	315	Jimma Town, Jimma, Oromia	Direct and formol-ether	586	134 (33.9%)	3
Gebrehiwot 2014 [104]	Cross sectional	Preschool children	195	179	WonjiShoa Sugar, Oromia region	Kato-Katz	374	91(24.3%)	2
Leta 2018 [89]	Cross sectional	School children	NA	NA	53 schools of Amhara region	Kato-Katz	2650	354 (13.4%)	3
King 2013 [42]	cross sectional	Both	1130	1228	South Gondar, Amhara region	Formol-ether	2338	1471(63%)	3
Mekonnen 2013 [90]	Clinical trial	School children	NA	NA	14 schools of Jimma town, Oromia region	Kato-Katz	840	437(52%)	3
Mahmud 2015 [47]	Clinical trial	School children	152	217	Mekele University, Tigray region	Direct, formal-ether and Kato-Katz	369	267(73%)	3
Mahmud 2013 [48]	cross-sectional	School children	288	312	Mekele, Tigray	Direct, formol-ether and Kato-Katz	600	421 (72%)	3
Tefera 2017 [72]	Cross sectional	School children	282	433	Mendera, Jimma, Oromia region	McMaster	715	346(48.4%)	2
Tefera 2015 [91]	Cross sectional	School children	364	280	Babble town, Harrerge, Oromia region	McMaster	644	89 (13.8%)	2
Nguyen 2012 [24]	Cross sectional	School children	341	323	AngolelaWoreda, Amhara region	Formal- ether	664	202(30.4%)	3
Hailu 2018 [92]	Cross sectional	School children	186	223	Bahir Dar, Amhara region	Formol-ether	409	237 (58%)	2
Beyene 2014 [21]	Cross sectional	School children	114	146	Jimma Health Center, Jimma, Oromia region	Direct and formol-ether	260	129 (49.6%)	3
Alemu 2011 [34]	Cross sectional	School children	157	162	Zarima town, Gondar, Amhara region	Direct and Kato-Katz	319	263 (82.4%)	3
Alemayehu 2015 [93]	Cross sectional	School children	201	183	DembaGirara, Woliata, South region	Direct and Kato-Katz	384	328 (85.4%)	1
Ali 1999 [94]	Cross sectional	School children	161	121	Asendabo Town, Jimma, Oromia region	Direct and Kato-thick	282	243(86.2%)	0
Tulu 2016 [68]	Cross sectional	School children	251	241	Birbir, Bale Zone, Oromia region	Direct and formol-ether	492	131(26.6%)	0
Unasho 2013 [73]	Cross sectional	School children	189	217	Gedeo, Woliata and Kambata and Amaro, South region	Direct	406	170 (41.9%)	0
Belyhun 2010 [53]	Follow up cohort	Preschool children	NA	NA	Butajira town, South region	Formol-ether	905	44 (4.9%)	3
Tulu 2014 [43]	Cross sectional	School children	172	168	Delo-Mena, Bale Zone, Oromia region	Direct and formol-ether	340	89(26.2%)	1
Erosie 2002 [44]	Cross sectional	School children	NA	NA	BolosoSorie, South region	Formol-ether	421	292(69.4%)	1
Tadesse 2005 [39]	Cross sectional	School children	271	144	Babile town, Harrerge, Oromia region	Formal ether	415	113(27.2%)	0
Adamu 2005 [52]	Cross sectional	Preschool children	149	147	Police hospital, Armed Forces General hospital, and Tikur Anbessa Hospital, Addis Ababa	Direct, formol-ether and Modified Ziehl-Neelsen	296	69(23.3%)	0
Jemaneh 1999 [95]	Cross sectional	School children	439	439	Gondar town, Gondar, Amhara region	Kato-Katz	878	437(49.7%)	0
Dejenie 2009 [60]	Cross sectional	School children	1012	998	Central Tigray, Tigray region	Direct	2000	571(28.6%)	0
Dejenie 2010 [69]	Cross sectional	School children	319	303	Tigray, Tigray region	Kato-Katz	622	165(26.5%)	0
Nyantekyi 2010 [14]	Cross sectional	Preschool children	140	148	Wondo Genet, South region	Kato-Katz and formal-ether	288	245 (85.1%)	1
Legesse 2010 [56]	Cross sectional	School children	167	214	Adama town, Oromia region	Kato-Katz and formol-ether	381	263 (69%)	0
Terefe 2011 [64]	Cross sectional	School children	218	201	Bushulo, Hawassa, South region	Kato-Katz	419	282(67.3%)	1
Assefa 2013 [59]	Cross sectional	School children	267	190	Suburbs of Mekelle city, Tigray region	Kato-Katz	457	109 (23.9%)	0
Debalke 2013 [96]	Cross sectional	School children	161	205	Jimma town, Oromia region	McMaster	366	166(45.4%)	1
Dejene 2008 [97]	Cross sectional	School children	481	319	Hintallo-Wejerat, Tigray region	Formal- ether	800	285(35.6%)	0
Fekadu 2008 [27]	Cross sectional	School children	63	37	Asendabo town, Jimma, Oromia region	Harada-Mori (Test tube culture)	100	66(66%)	0
Haileamlak 2005 [33]	Cross sectional	Preschool children	487	437	Jimma Zone, Jimma, Oromia region	Direct and formal-ether	924	530(57.4%)	1
Jemaneh 2001 [46]	Cross sectional	School children	282	405	Chilga, Gondar Zone, Amhara region	Kato-Katz	687	470(68.4%)	1
Firdu 2014 [71]	Case-control	Both	135	95	Yirgalem Hospital, South region	Direct formol-ether and modified Ziehl-Neelsen	230	74(32.2%)	1
Wale 2014 [65]	Cross sectional	School children	206	196	Lumame town, Amhara region	Direct and formal ether	402	219(54.5%)	1
Teklemariam 2014 [70]	Cross sectional	School children	252	228	Enderta, Tigray region	Formalin-ether	480	199(41.5%)	0
Ayalew 2011 [36]	Cross sectional	School children	358	346	Delgi, Gondar, Amhara region	Direct and formol-ether	704	562 (79.8%)	2
Merid 2001 [38]	Cross sectional	School children	NA	NA	Lake Hawassa, South region	Direct and formol- Ether	150	139(92.7%)	0
Assefa 1998 [30]	Cross sectional	School children	479	219	Wollo, Amhara region	Formal-ether	698	304 (43.3%)	0
Roma 1997 [50]	Cross sectional	School children	352	168	Wondo-Genet, South region	Formol-ether	520	465 (89.4%)	1
Abera 2013 [98]	cross sectional	School children	397	381	Bahir Dar special zones, Amhara region	Kato-Katz and formal-ether	772	401(51.5%)	3
Kidane 2014 [55]	Cross sectional	School children	177	207	Wukrowereda, Tigray region	Direct	384	233(60.7%)	0
Alamir 2013 [29]	Cross sectional	School children	192	207	Dagi, Amhara region	Direct and formal-ether	399	311(77.9%)	0
Kabeta 2017 [99]	Cross sectional	Preschool children	NA	NA	HawassaZuria, South region	Direct smear and formal-ether	587	301(51.3%)	1
Shumbej 2015 [100]	Cross sectional	Preschool children	165	212	Butajira, South region	McMaster	377	104 (27.6%)	3
Tadege 2017 [101]	Cross sectional	School children	235	139	Finchawa and Tullo, South region	Formol-ether	374	254(67.9%)	3
Andualem 2014 [102]	Cross sectional	School children	168	190	Motta, Gojam, Amhara region	Direct and formal-ether	358	245(68.4%)	0
Reji 2011[51)	Cross sectional	School children	NA	NA	Adama town, Oromia region	Kato-Katz	358	127 (35.5%)	1
Alemu 2014 [67]	Cross sectional	School children	211	194	Umolante, GamoGofa, South region	Kato-Katz	405	109(26.9%)	0
Samuel 2015 [105]	Cross sectional	School children	NA	NA	Ambo town, Oromia region	Formol-ether	375	47(12.6%)	3
Teshale 2018 [103]	Cross section	School children	240	170	MedebayZana, Tiray region	Kato-katz	410	52(12.7%)	1
Tekeste 2013 [63]	Cross sectional	School children	170	156	Gorgora, Amhara region	Kato-katz	326	110(36.8%)	2

Abbreviations: *NA* not available; *PCR* Polymerase chain reaction

**Fig. 2:**
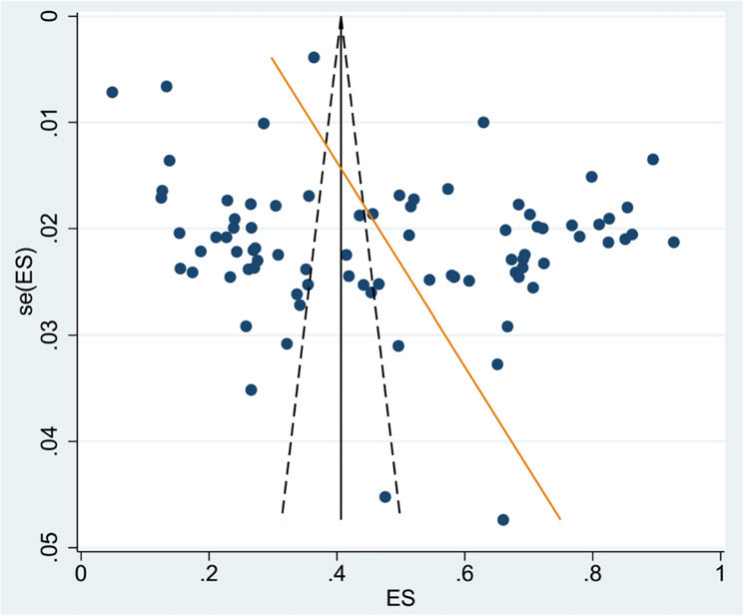
Begg’s funnel plot and Egger test for heterogeneityof intestinal parasite infections among Ethiopian children

**Fig. 3:**
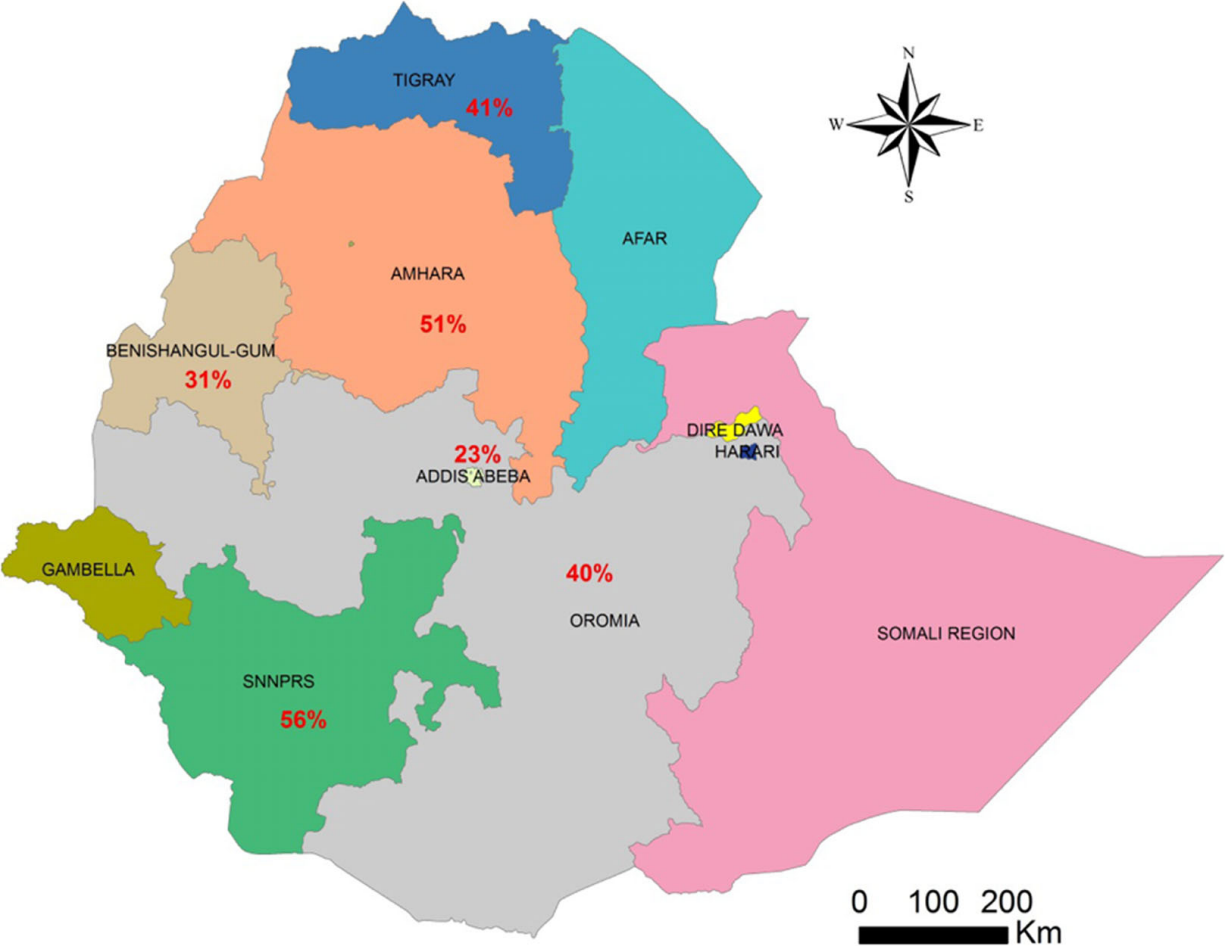
Regional distribution of intestinal parasite infections in Ethiopian children from 1997 to 2019

**Fig. 4:**
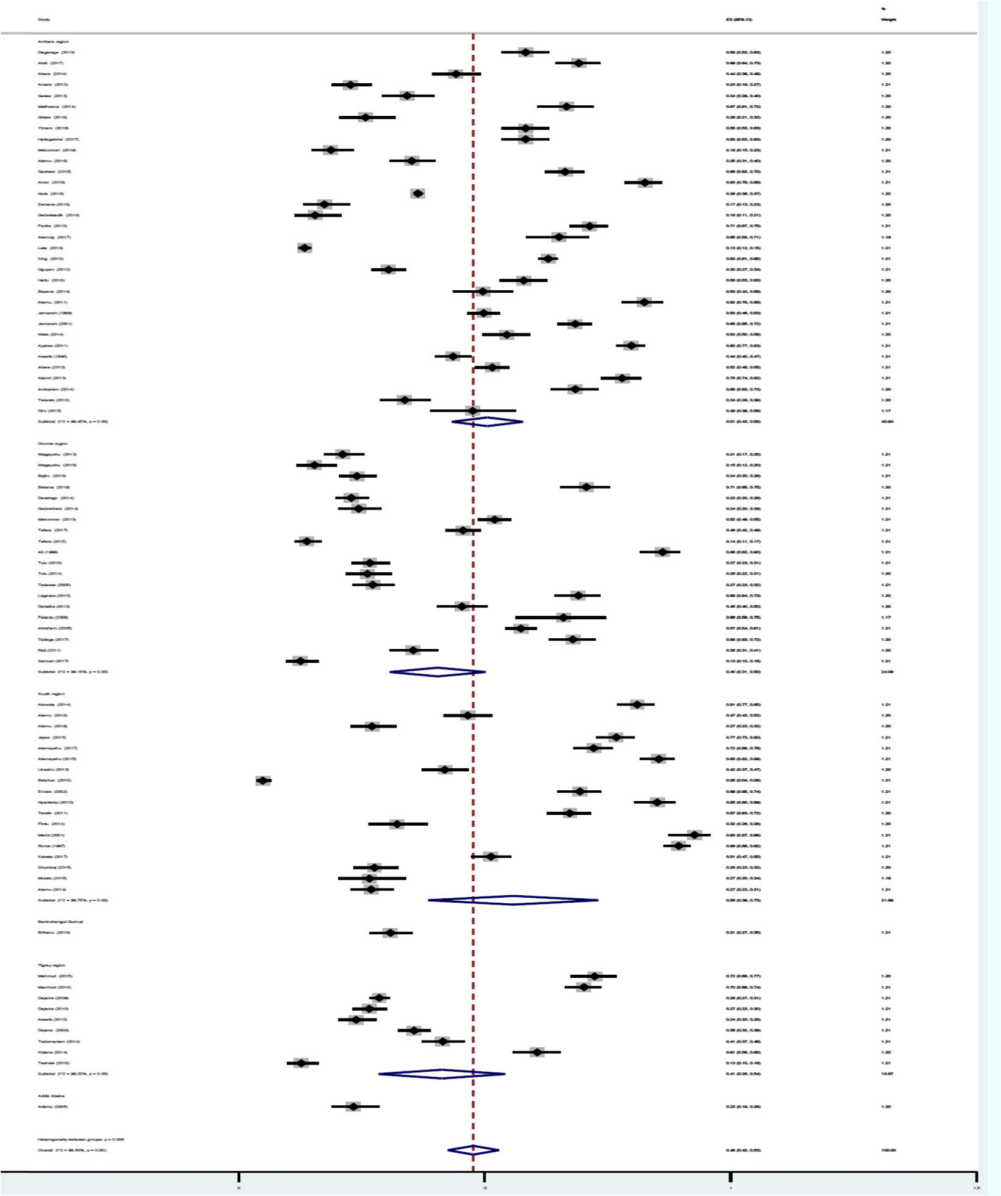
Forest plot showing the geographic distribution of intestinal parasite infections in Ethiopia Children

**Fig. 5:**
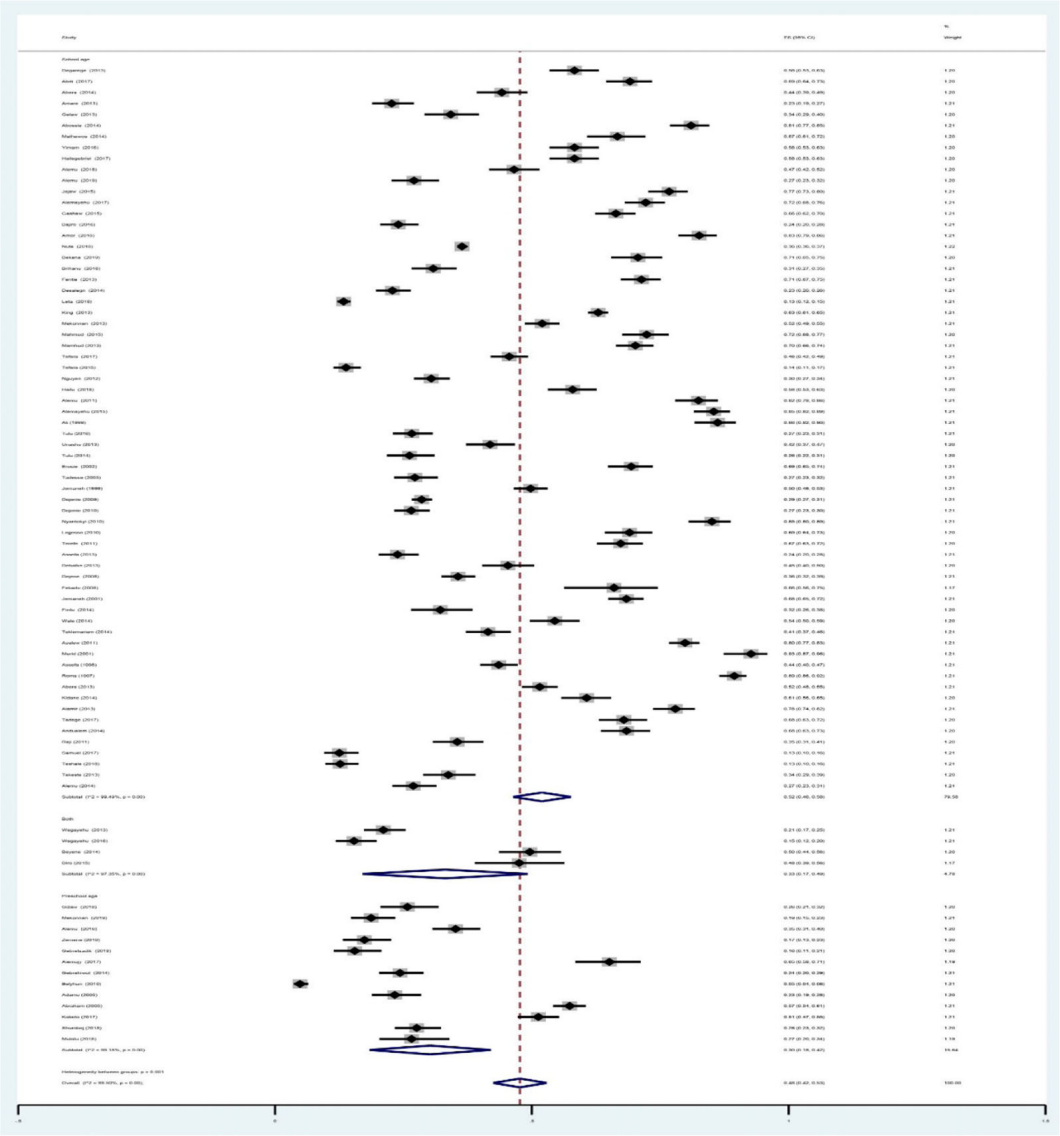
Forest plot showing age related distribution of intestinal parasite infections in Ethiopia children

A total of 27,354 of the 56,786 children examined during the period under review were infected with one or more species of intestinal parasites yielding an overall prevalence of (*n* = 27,354) 48%(95%CI: 42 to 53%) with substantial heterogeneity (I^2^ = 99.50%, regression coefficient: -0.23, 95% CI: − 0.38 to − 0.09, *p* = 0.002, Fig. 2). A range of parasites were detected in the studies including *Ascaris lumbricoides, Hookworm, Trichuris trichuria, Strongyloides stercoralis, Enterobius vermicularis, Schistosoma mansoni, Hymenolepsis nana, Taenia species, Giardia lamblia/intestinalis/duodenalis, Entamoeba histolytica/dispar and Cryptosporidium species*. Subgroup analysis showed that the prevalence of IPIs was 56% (95%CI: 39 to 73%) in Southern region, 51%(95%CI: 43 to 58%) in Amhara region, 40% (95%CI: 31 to 50%) in Oromia region, and 41%(95%CI: 28 to 54%) in Tigray region as shown in Figs. 3 and 4. The age related prevalence was 52% (95%CI: 46 to 58%,) in school-age children and 30% (95%CI: 18 to 34%) preschool-age children (*p* = 0.002) as shown in Fig. 5.

**Fig. 6:**
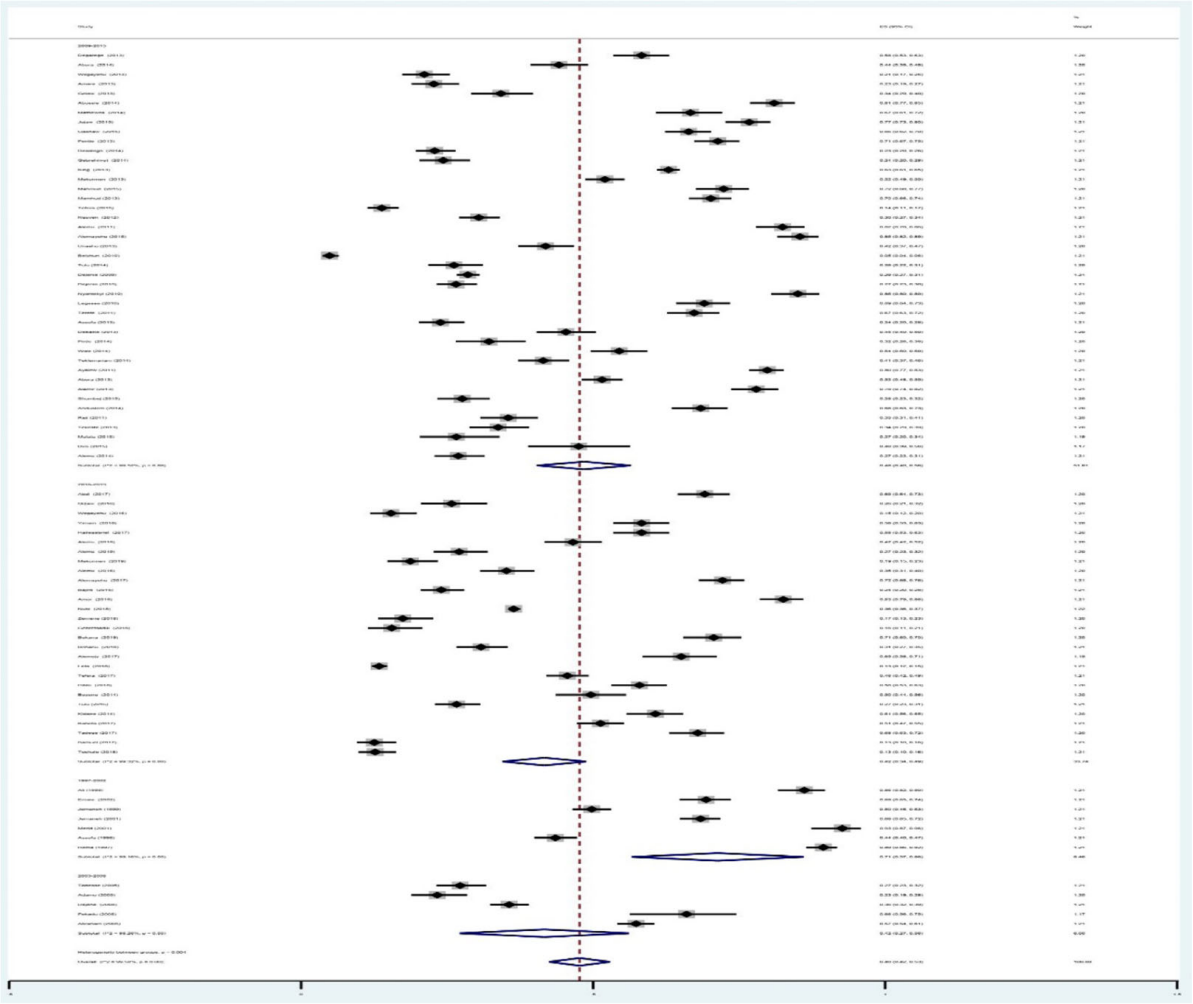
Forest plot showing trend of intestinal parasite infections in Ethiopia children

**Fig. 7:**
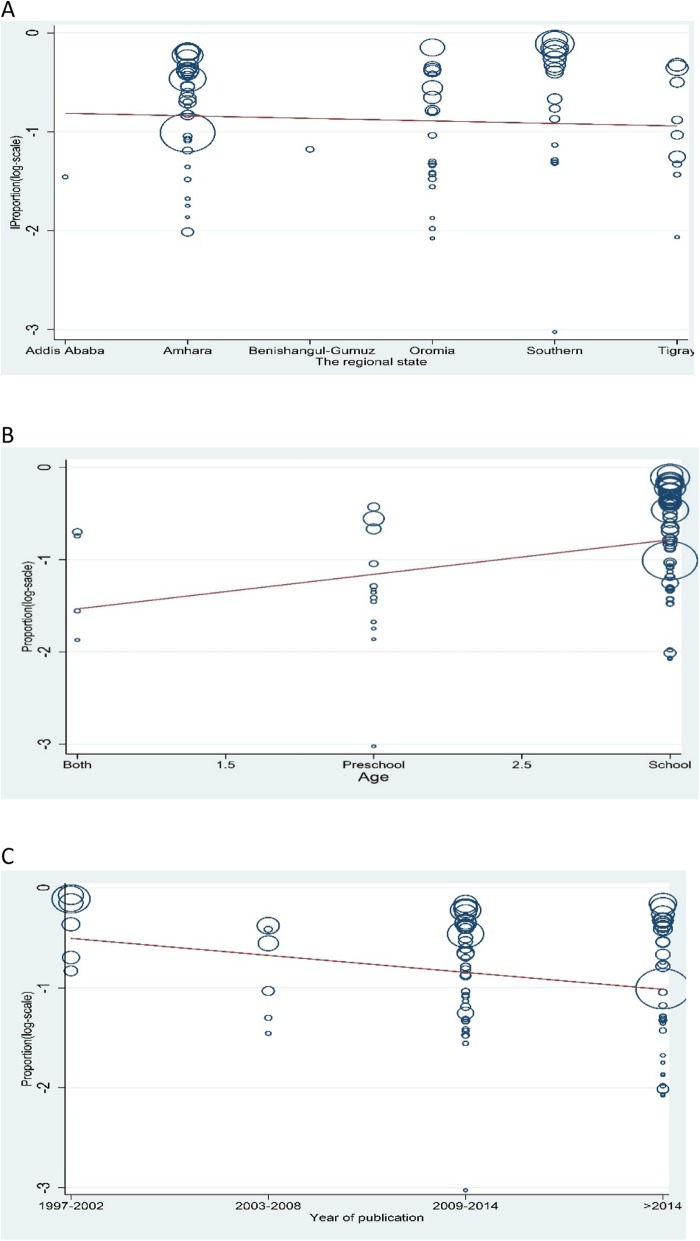
Meta regression result of (**a**). the geographic distribution (**b**). the distribution by age C. distribution by year of publication of Intestinal parasite infections among Ethiopian children

The pooled prevalence of IPIs in year 1997–2002, 2003–2008, 2009–2014 and > 2014 was 71% (95% CI: 57 to 86%), 42% (95% CI: 27 to 56%), 48% (95% CI: 40 to 56%) and 42% (95% CI: 34 to 49%), respectively [Fig. 6]. We did meta-regression analyses to search for the sources of heterogeneity. We detected no significance difference in geographical distribution (regression coefficient: 0.025, 95% CI: − 0.11 to 0. 06, *p* = 0.56) as shown Fig. 7a.The results of the analyses showed that age (regression coefficient: 0.38, 95% CI: 0.15 to 0.60, *p* = 0.002, Fig. 7b) and year of publication (regression coefficient: -0.17, 95% CI: − 0.32 to − 0.025, *p* = 0.023, Fig. 7c) might be sources of heterogeneity,

### Prevalence of IPIs by area of residence, gender and poly-parasitism status

Thirteen studies (*N* = 12,356) reported the proportion of IPIs based on residence area. The pooled prevalence of overall IPI was not significantly differ between rural and urban areas; rural 22 95% CI: 10 to 30%, Additional file 1) and urban 23% (95% CI: 14 to 32%, Additional file 2). Forty two studies (*N* = 36,218) had separate data on the prevalence of IPIs for males and females. The pooled prevalence formales was 24% (95%: CI 20 to 28%, Additional file 3) while, it was 22% (95% CI: 18 to 25%, Additional file 4) for females. Poly-parasitism was observed in 16% (95% CI: 13 to 19%, Additional file 5) of children and 36% (95% CI: 30 to 41%, Additional file 6) of children were infected with a single species of parasite.

## Discussion

The pooled prevalence of IPIs in preschool and school-age Ethiopian children was 48%(95%CI: 42 to 53%). The prevalence is higher in Southern (56%) and Amhara regions (51%).We observed a significant decrease in the prevalence of IPIs among children in Ethiopia over the last two decades (22 years). The burden of infection was higher among school-age children compared to preschool-age children (52% vs.30%, *p* = 0.002), however, it was similar in males and females as well as in urban and rural inhabitants. Poly-parasitism was observed in 16% of preschool and school-age children while, single infection was documented in 36% of the children participated in the study.

The overall pooled prevalence estimate (48%) observed in the present systematic review and meta-analysis is similar to the study from Nigeria (54.8%) [106], Rwanda (50.5%) [107], Afghanistan(47.6%) [108],Syria (42.5%) [109] and in Palestine (40.5%) [110]. However, the finding of this systematic review and meta-analysis is higher than that of Cameron(24.1%) [111], Rwanda (25.4%) [112], Iran (38%) [113], Turkey(31.7–37.2%) [114] and Egypt (26.5%) [115]. The difference might be attributed to socio-economic status, poor hygiene and sanitary facilities, weather, climate and environmental factors. For example, a study in Ethiopia showed that *Ascaris lumbricoides*infections were more common in children living in households with lower incomes (prevalence ratio = 6.68, 95% CI = 1.01–44.34) and that *Giardia lamblia* infections were more common in children living in households that used an unprotected water source (prevalence ratio = 1.95, 95% CI = 0.96–3.99) [32]. In addition, most Ethiopian communities have the habit of consuming uncooked meat, which mightincrease the risk of exposure to human helminths. Many of Ethiopian population where the studies conducted involved in irrigation activities for the cultivation of vegetables during the dry season. This irrigation canals create media for the reproduction of vector snails, which might be the cause of the appearance of endemicity of Trematodes infections in the area. It might also be attributed to the specificity and sensitivity of the diagnostic methods employed by the individual studies.

The meta-regression of prevalence of IPIs over time showedsignificant decreasing trends in each 6-years block by 17% (95% CI: 2.5 to 32%) and this declined prevalence was probably due to socioeconomic development, improvement in sanitation and large-scale deworming programs. Many studies from around the world have reported a significant decreasing trend in the prevalence of overall IPIs in recent years,such as the global burden of disease study [5], study from Burkina Faso [116], Nepal [117], Brazil [118] and other from 43 Sub-Saharan [119]. Despite many initiatives and efforts to introduce mass deworming program and improvement in water quality and sanitation, IPIs are still prevalentand the decrease in trend is less than that of other countries (Ethiopia 42% in 2016–2019 vs. Nepal 20. 4% in 211–2015 and Brazil 23.8% in 2010–2011). This might be possibly due to insufficient financial supports in implementation of the strategies that have been known to reduce the infection such as access to safe water supply, personal hygiene and sanitation, deworming and public health awareness.. In addition, lack of political commitment, social and environmental factors might also contribute for the higher prevalence of IPIs in the country. Inadequate community involvement and ownership of control activities are also another possible reason.

The prevalence of IPIs in school-age children was (52%), which was significantly higher than in preschool-age children (30%). This is similar to the study by Jayarani 2014 [120] and Workneh 2014 [121], but opposite to the study by Daryani 2017 [113]. School children carry the heaviest burden of the intestinal parasite associated morbidity due to their habits of playing or handling of contaminated soils, eating with soiled hands, unhygienic toilet practices, drinking and eating of contaminated water and food [22] compared to preschool-age children who usually cared by families. The current control efforts in Ethiopia usually target school-age children, but a significant proportion of preschool-agechildren (30% in this study) were also infected and can be source for the re-infection of treated school-age children. Therefore, it worthy revising the national control program based on regional and national prevalence which included preschool children and other population at risk.

In the present study, the prevalence of IPIs in females (22%) was similar to males (24%), which is similar to the study by Gelaw 2013 [45], but in contrast to study by Daryani 2017 [113] in Iran. In Iran, report indicated that more females have (30.9%) have IPIs than males (16.5%). The difference might be due to cultural and behavioral difference between the two countries.

The distribution of IPIs in this study was relatively similar in both urban and rural areas. This might be due to absence of proper human waste disposal systems, the shortage of safe water supply, the social and poor environmental or personal hygiene in many unplanned urban areas in Ethiopia in addition to similarity of eating habit and life style of both urban and rural areas of the country. So far, reports from Africa and South Asia countries are conflicting. Some were reported higher infection rates of IPIs in rural areas compared to urban areas [122–126] and others reported higher rate of infections in urban children [127]. In fact, comparable data on IPIs in urban and rural settings are very limited. For instance, only 13 studies out of 83 studies included in this meta-analysis were reported prevalence of IPIs in both urban and rural areas and therefore, indicating more work to be done in the future to resolve this issue.

We estimated the geographical distribution and identified high risk areas that should be prioritized control interventions, which complement global efforts towards elimination of IPIs infections by 2020. In addition, this work also highlighted the need for survey in areas where data are not available such as Somalia region, Afar region, Harari, Dire Dawa city and Gambella region or scarce (Addis Ababa city and Benishangul-Gumuz region). The essence of current systematic review and meta- analysis of IPIs data analysis among preschool and school-age children in Ethiopian were to support the efforts undertaken to control and eliminate neglected tropical diseasesby nurturing or supplementing useful national epidemiological data. We hope that the findings of current study provide valuable information to the policymakers, National Health Bureau and other concerned bodies about national and regional distribution and their prevalence in Ethiopia preschool and school-children.

There are a few limitations of the present meta-analysis, which may affect the results. First of all, the review protocol is not registered which could be source of bias.. It is prudent to interpret the results of this study as 37(44.6%) of the included studies were low quality based on our quality assessment criteria. In all of studies included in this review, single stool sample examination were used despite multiple stool samples recommendation for standard diagnosis and therefore, possible underestimation of the prevalence. There is also substantial heterogeneity observed between the studies that affect the interpretation of the results. However, we did meta-regression analyses on various sources including geographical distribution, age category and year of publication. These might comefrom age category (*P* = 0.002) and year of publication (*P* = 0.023) but not from geographic distribution (*p* = 0.56).

## Conclusion

Intestinal parasite infections are highly prevalent and well distributed across the regional states of Ethiopia. Southern and Amhara regional states carry the highest burden. Although school-age children have higher prevalence of IPIs compared to preschool-age children, the prevalence is still unacceptably higher among preschool-age children. We observed a gradual, but significant decrease in prevalence of IPIsamong preschool and school-agein Ethiopian in the last two decades with no significant difference between males and females. The prevalence in the most recent 6 years was around 42% compared to 71% in the late 1990s.Place of residence has no effect on the burden of IPIs among preschool and school-age in Ethiopian. Sixteen percent (16%) of preschool and school-age children had concurrent poly-parasitism infections.

## Supplementary information

**Additional file 1.** Forest plot showing prevalence of intestinal parasite infections among rural preschool and school-age children in Ethiopia.

**Additional file 2.** Forest plot showing prevalence of intestinal parasite infections among Urban Ethiopia children.

**Additional file 3.** Forest plot showing prevalence of intestinal parasite infections among male Ethiopian children.

**Additional file 4.** Forest plot showing prevalence of intestinal parasite infections among female Ethiopian children.

**Additional file 5.** Forest plot showing prevalence poly-parasitism infections among Ethiopian children.

**Additional file 6.** Forest plot showing prevalence intestinal parasite infections with single species of parasite among Ethiopian children.

## Data Availability

The datasets used and/or analyzed during the current study available from the corresponding author on reasonable request.

## Abbreviations

CI: Confidence interval
GRADE: Grading of Recommendations Assessment, Development and Evaluation
IPIs: Intestinal parasite infections
MDA: Mass drug administration
NGOs: Non-governmental organizations
PRISMA: Preferred Reporting Items for Systematic Reviews and Meta-Analyses
STHs: Soil-transmitted helminths
