# Supplementary material for: Epidemiology of intestinal parasitic infections in preschool and school-aged Ethiopian children: a systematic review and meta-analysis
Source: BMC Public Health. 2020 Jan 28;20:117. doi: 10.1186/s12889-020-8222-y (PMC6988312; doi:10.1186/s12889-020-8222-y)
Supplement: Supplementary file 5 — Additional file 5. Forest plot showing prevalence poly-parasitism infections among Ethiopian children. [file 12889_2020_8222_MOESM5_ESM.docx]

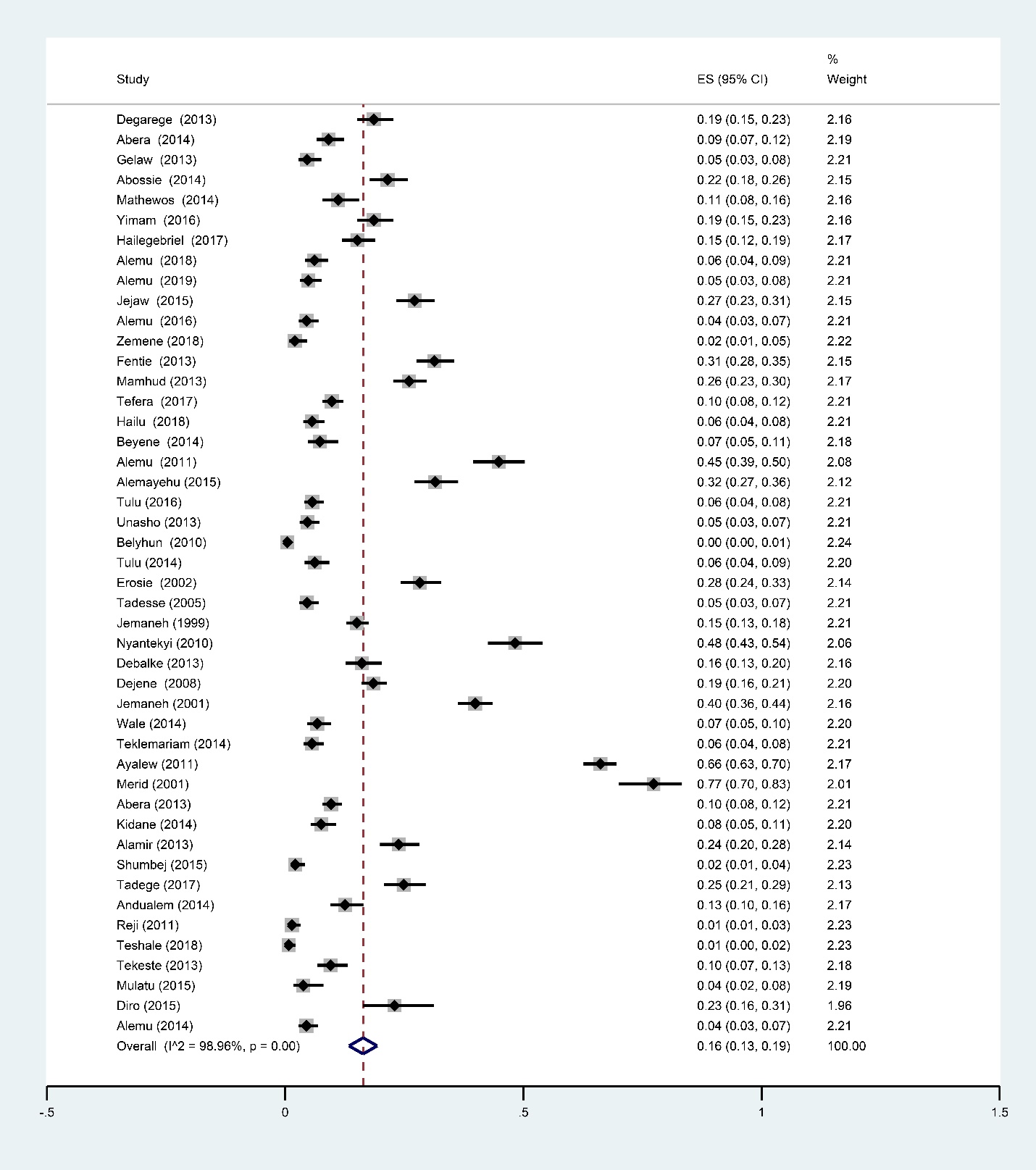


Additional file 5. Forest plot showing prevalence poly-parasitism infections among Ethiopian children
